# A randomised trial of observational learning from 2D and 3D models in robotically assisted surgery

**DOI:** 10.1007/s00464-018-6203-3

**Published:** 2018-05-14

**Authors:** David J. Harris, Samuel J. Vine, Mark R. Wilson, John S. McGrath, Marie-Eve LeBel, Gavin Buckingham

**Affiliations:** 1 Sport and Health Sciences, University of Exeter, Exeter, UK; 2 Exeter Surgical Health Services Research Unit, RD&E Hospital, Exeter, UK; 3 University of Exeter Medical School, Exeter, UK; 4 Division of Orthopaedic Surgery, Western University, Ontario, Canada

**Keywords:** Observational learning, Robotically assisted surgery, 3D, Stereoscopic, Surgical training

## Abstract

**Background:**

Advances in 3D technology mean that both robotic surgical devices and surgical simulators can now incorporate stereoscopic viewing capabilities. While depth information may benefit robotic surgical performance, it is unclear whether 3D viewing also aids skill acquisition when learning from observing others. As observational learning plays a major role in surgical skills training, this study aimed to evaluate whether 3D viewing provides learning benefits in a robotically assisted surgical task.

**Methods:**

90 medical students were assigned to either (1) 2D or (2) 3D observation of a consultant surgeon performing a training task on the daVinci S robotic system, or (3) a no observation control, in a randomised parallel design. Subsequent performance and instrument movement metrics were assessed immediately following observation and at one-week retention.

**Results:**

Both 2D and 3D groups outperformed no observation controls following the observation intervention (*p*s < 0.05), but there was no difference between 2D and 3D groups at any of the timepoints. There was also no difference in movement parameters between groups.

**Conclusions:**

While 3D viewing systems may have beneficial effects for surgical performance, these results suggest that depth information has limited utility during observational learning of surgical skills in novices. The task constraints and end goals may provide more important information for learning than the relative motion of surgical instruments in 3D space.

Observational learning benefits skill acquisition across both simple and complex motor tasks [1, 2] and plays an important role in surgical skills training, especially in novice learners [3]. Research suggests that observing others provides information about specific features of the model’s action and constraints of the task [1], allowing a blueprint of the ideal action to be built [4]. Many observational learning interventions, however, have utilised videos rather than live observation in an attempt to standardise models. This means that the additional information provided by three dimensional (3D) images, such as distance between objects in depth, is not present [5, 6]. Advances in surgical simulation using 3D and virtual reality technology mean that video models can now be viewed in three dimensions, in closer correspondence with the action reproduction. Whether this additional depth information actually benefits surgical skill learning is yet to be empirically tested.

Seeing the world in three dimensions requires the visual system to reconstruct the 3D configuration of objects from a pair of two dimensional (2D) retinal images. A range of monocular depth cues are provided by the environment [7–9], but 3D perception can be elicited purely by binocular disparity, from the slight difference in position and orientation of corresponding images entering the left and right eye [10, 11]. This effect is mimicked by modern surgical systems providing a 3D image via the presentation of separate images to the left and right eyes. The daVinci robotic platform used in the current study relays 3D information from two HD endoscope cameras to the operating console using this method. In minimally invasive procedures, surgeons have traditionally relied upon experience and monocular depth cues to interpret the image from a 2D endoscope [12], but there is emerging evidence that 3D systems are providing benefits for surgical performance [13–15]. Yet it remains unclear whether 3D observation provides similar benefits for motor skill learning, such as acquiring proficiency with laparoscopic or robotic instruments.

While observing an expert model is a powerful tool for learning basic surgical motor skills, opportunities to do this in the operating room are somewhat limited. The use of simulators and e-learning provides trainees with greater possibilities for observing surgical procedures, but inferring 3D information indirectly from a 2D image could lead to impoverished learning. Given that the reproduced movement must be made across three dimensions, observed movements might be better understood when 3D information is present [16]. It is, therefore, important to understand whether 3D viewing technology can benefit observational learning of surgical skills, over and above 2D viewing.

As 3D viewing benefits visual guidance of action [17], and may improve surgical performance [13, 14], we aimed to evaluate whether *observing* a 3D video model would benefit skill acquisition in a robotically assisted surgical task. As binocular vision has been found to improve performance and alter movement kinematics in reaching tasks [6, 17], it was predicted that a 3D model would promote more effective learning than a 2D model or no-observation control, immediately following an observation intervention and at 1-week follow-up.

## Materials and methods

### Participants

90 undergraduate medical students (34 males, mean age = 19.9 years, SD = 2.6) were recruited from the University of Exeter Medical School and assigned to groups in a randomised parallel design. Participants had no previous experience of robotically assisted surgery. The sample size was based on previous research investigating observational learning in a surgical simulation task [18]. University ethical approval was acquired prior to data collection. Participants gave written informed consent at the start of testing and were compensated £15 for participation.

### Task and equipment

**Fig. 1:**
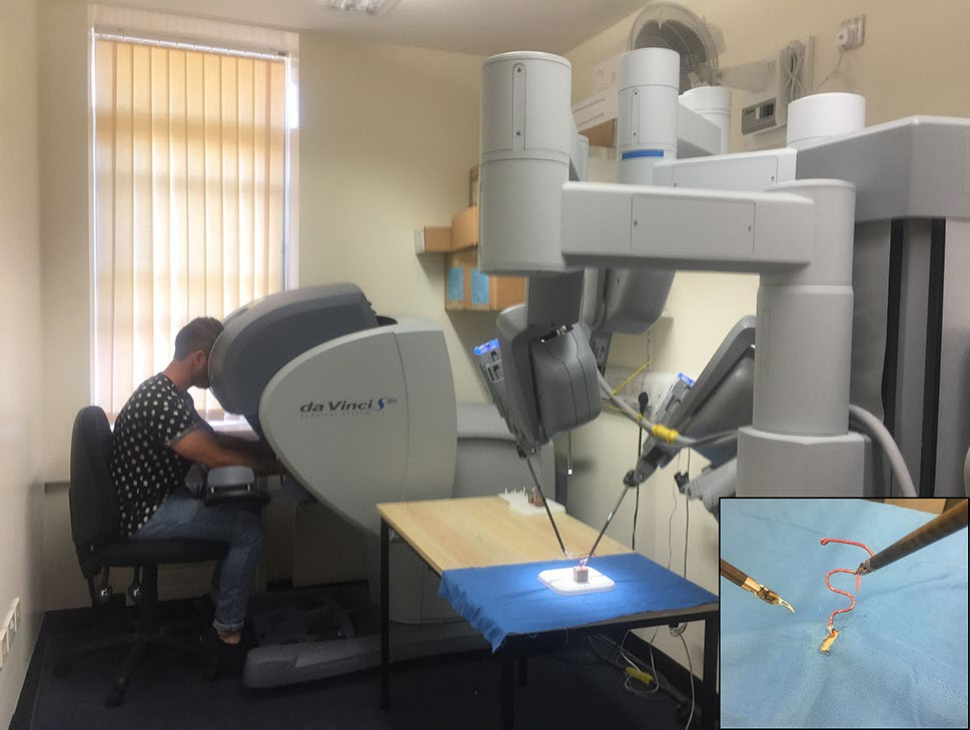
The daVinci S system, with ring-carrying task (inset)

Participants performed a ring tower transfer exercise adapted from a surgical skills training curriculum (Fundamentals of Robotic Surgery) [19]. The aim of the task is to carry a red ring along a curved wire from the base to the end of the wire with the right hand, transfer it into the left hand and then return to the starting position without touching the wire. This task was chosen to assess proficiency of instrument control (Fig. 1 inset).

The ring task was carried out on the daVinci S robotically assisted surgical platform (Intuitive Surgical Ltd.). The system consists of a 3D viewing control console and a separate operating cart with three robotic arms, two carrying laparoscopic tools (a needle driver with articulated wrist) and one carrying an endoscope (Fig. 1). The instruments are fingertip-controlled with surgical instruments mimicking the movements of the surgeon’s hand (forefinger and thumb) and wrist.

### Training videos

The 3D expert video was recorded in high definition (HD) on GoPro Hero 3+ cameras using the GoPro dual hero system, which time locks two side-by-side cameras, with the videos combined into a stereoscopic video in GoPro CineForm Studio. The video featured a consultant urologist experienced with the daVinci system performing the ring-carrying task. The 2D condition used the 2D, non-stereoscopic version of the same HD video.

### Procedure

Participants were required to attend two testing sessions (approximately 30 min each) at the Surgical Health Service Research Unit (HeSRU) of the Royal Devon and Exeter Hospital. Visits were separated by approximately 7 days. At visit one, participants performed a baseline test on the ring-carrying task, followed by the video intervention and a post-test. Visit two assessed retention of learning on the ring-carrying task.

At the start of testing, participants observed a video explaining the experimental procedures and were tested for stereoscopic acuity (1–10 scoring) using the Randot stereoacuity test (Stereo Optical Inc., Chicago, IL). Participants then had one minute to familiarise themselves with the console controls as in [20]. Experimental task performance was recorded for later analysis using a GoPro Hero 3+ camera positioned out of view. GENEActiv accelerometers, which record tri-axial accelerations at 100 Hz, were attached to the robot arms to record instrument movements.

Following the baseline test, participants were randomly assigned to one of three observation conditions (3D, 2D or no observation), based on random number generation. Participants in the 3D group watched two run-throughs of the ring transfer task (lasting 130 s), performed by an expert surgeon, displayed in 3D, while the 2D group watched an identical 2D high definition video. No observation controls were given a simple wordsearch puzzle for a matched time period. The 3D video was displayed using Nvidia 3D vision two wireless glasses (Nvidia, Santa Clara CA), which use an infrared connection to the PC to synchronise 120 Hz shuttering between the glasses and monitor, creating stereoscopic images. Both videos were presented on a 3D vision ready, 24 in. Acer monitor, while participants positioned their head in a chin rest 48 cm from the screen. An active 3D system was chosen based on the available technology, although many newer surgical systems use passive 3D for greater wearer comfort [15].

### Data analysis

Time to completion was first obtained from the video recording of the experimental task. Secondly, the number of errors was counted from the video footage. Errors were scored based on their severity: small touches of the wire scored one point and a drag along the wire or dropping the ring scored two points. The number of errors was divided by the time to completion to determine the rate of errors—our primary dependent variable.

Accelerometry data were downloaded and synchronised using GENEActiv PCSoftware, and then pre-processed using a custom-written Matlab script. Raw data were filtered using a 2nd order, 3 Hz Butterworth low pass filter to reduce noise [21], before converting to the Euclidean Norm Minus One (ENMO), which integrates *x, y* and *z* plane accelerations and removes the net effect of gravity (1 g) [22]. Matlab was also used to calculate jerk (derivative of accelerations with respect to time), using the formula Jerk = ΔAcc/ΔTime. Sample entropy [23] of accelerations, which indicates a lack of regularity, was calculated from the natural logarithm of the conditional probability that a series similar for *n* points remains similar at the next point.

Statistical analysis was performed using JASP version 0.8.5.1 [24]. Outliers were identified (± 3 SD from the mean), with three performance scores removed (two from the 3D group and one from the 2D group, 1%) and three accelerometry scores removed (all from the 3D group; < 1%). Dependent variables were analysed using separate 3 (trial) × 3 (group) mixed ANOVAs, with Bonferroni-Holm corrected post hoc tests where appropriate. Data was checked for homogeneity of variance (Levene’s test), and skewness and kurtosis coefficients indicated the data to be normally distributed. Violations of sphericity were corrected for using a Greehouse-Geisser correction factor. Bayes Factors (BF_10_) for main effects and post hoc tests were also obtained using a symmetric Cauchy prior. Our raw data are available from the Open Science Framework (https://osf.io/n324a/).

## Results

Group comparisons indicated no group differences in age (*p* = 0.10, BF_10_ = 0.64) or stereo acuity (*p* = 0.30, BF_10_ = 0.26), showing the groups to be well matched.

In order to evaluate the effect of the observation condition on performance, a 3 (trial) × 3 (group) mixed ANOVA was conducted on the rates of errors. There was a significant main effect of trial, F(1.65,142.28) = 10.29, *p* < .001, *ω*^2^ = 0.104, BF_10_ = 351.75. There was no significant effect of group, F(2,86) = 2.92, *p* = 0.06, *ω*^2^ = 0.064, BF_10_ = 1.18, and no interaction between the variables, F(3.31,142.28) = 1.27, *p* = 0.29, *ω*^2^ = 0.026, BF_10_ = 0.13. Bonferroni-Holm corrected *t*-tests showed a significant performance improvement from baseline to post-intervention (*p* < 0.001, BF_10_ = 106.20), and baseline to retention (*p* = 0.003, BF_10_ = 15.92), but not from post-intervention to retention (*p* = 0.68, BF_10_ = 0.13).

As the omnibus group differences approached significance, one-way ANOVA was used to compare group differences at each time point. At baseline, there were no significant group differences, *F*(2,87) = 0.92, *p* = 0.40, *ω*^2^ = 0.000, BF_10_ = 0.21. A significant effect of group was found post-intervention, *F*(2,87) = 4.40, *p* = 0.02, *ω*^2^ = 0.070, BF_10_ = 3.12. Bonferroni–Holm corrected *t*-tests showed both 2D (*p* = 0.03, BF_10_ = 2.19) and 3D (*p* = 0.03, BF_10_ = 1.82) groups performed better than controls, but there was no difference between 2D and 3D (*p* = 0.84, BF_10_ = 0.28). At retention, however, there was no significant group difference in performance, *F*(2,86) = 1.78, *p* = 0.18, *ω*^2^ = 0.017, BF_10_ = 0.42.

In order to evaluate the effect of the observation condition on instrument movements, 3 (trial) × 3 (group) mixed ANOVAs were conducted on accelerometry measures. For average jerk, there was a significant main effect of trial, *F*(1.65,143.41) = 6.40, *p* = 0.004, *ω*^2^ = 0.057, BF_10_ = 11.94. There was no significant effect of group, *F*(2,87) = 0.34, *p* = 0.72, *ω*^2^ = 0.000, BF_10_ = 0.24, and no interaction, *F*(3.30,143.41) = 0.64, *p* = 0.61, *ω*^2^ = 0.000, BF_10_ = 0.06. Bonferroni–Holm corrected *t*-tests indicated a significant increase in jerk from post-intervention to retention (*p* < 0.001, BF_10_ = 273.04), but there was no difference between baseline and post-intervention (*p* = 0.27, BF_10_ = 0.21) or baseline and retention (*p* = 0.09, BF_10_ = 0.89).

For acceleration entropy, there was a significant main effect of trial, *F*(1.70,148.14) = 11.86, *p* < 0.001, *ω*^2^ = 0.109, BF_10_ = 1298.75. There was no significant effect of group, *F*(2,87) = 0.31, *p* = 0.73, *ω*^2^ = 0.000, BF_10_ = 0.16, and no interaction, *F*(3.41,148.14) = 0.36, *p* = 0.81, *ω*^2^ = 0.000, BF_10_ = 0.04. Bonferroni–Holm corrected *t*-tests indicated a significant decrease in entropy from baseline to post-intervention (*p* = 0.003, BF_10_ = 14.27), from baseline to retention (*p* < 0.001, BF_10_ = 382.49), and from post-intervention to retention (*p* = 0.02, BF_10_ = 1.39).

## Discussion

The aim of this study was to compare observational learning from a 2D versus a 3D model. Observational learning plays an important role in surgical training [25, 26] and recent developments in surgical simulation and virtual reality mean that surgeons can be trained in 3D environments that mimic the 3D displays of robotic surgical systems. Evidence suggests that depth information may have beneficial effects on surgical performance [12, 14, 27], but it is unclear whether 3D observation provides a more effective model to learn from.

**Fig. 2:**
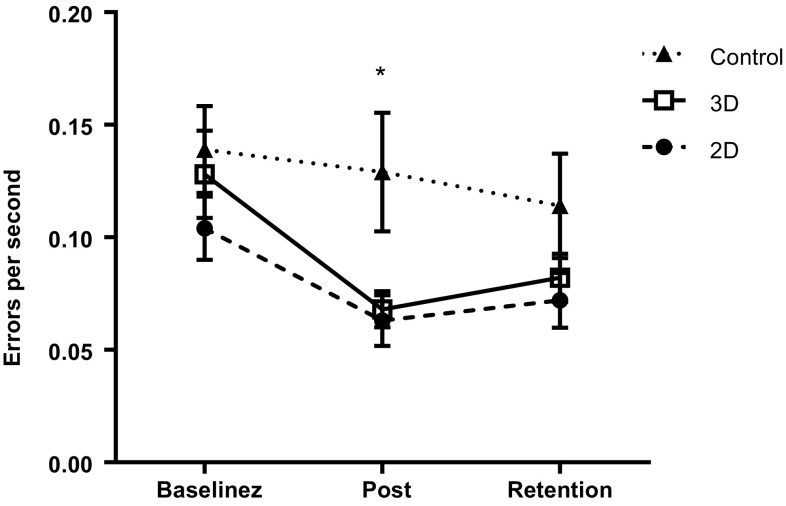
Mean (± S.E.M.) 2D, 3D, and control group performance across testing conditions (*p < 0.05)

**Fig. 3:**
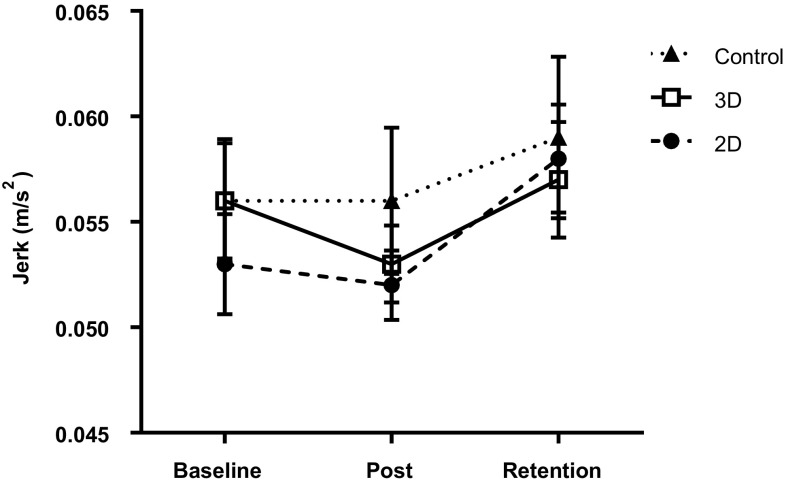
Mean (± S.E.M.) instrument jerk in 2D, 3D, and control groups across testing conditions

**Fig. 4:**
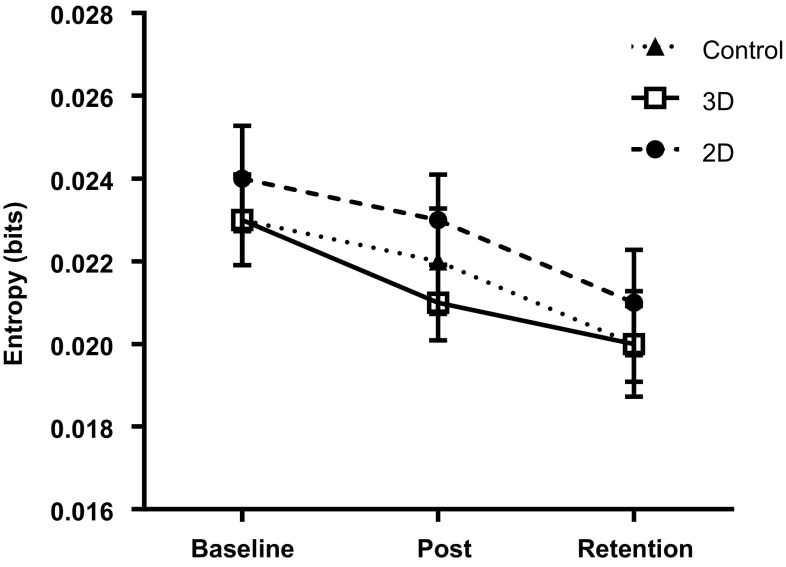
Mean (± S.E.M.) acceleration entropy in 2D and 3D groups across testing conditions

Contrary to our hypothesis, there was no evidence that 3D observation was more beneficial for learning this particular task than 2D observation. Across the three groups, error rates improved from baseline (pre-observation) to post-intervention, and this improvement was largely retained at a one-week follow-up (Fig. 2). Greater learning occurred in the observation groups, with 2D and 3D groups significantly outperforming non-observation controls post-intervention. However, there was no difference in performance between 2D and 3D observation immediately post-intervention or at retention. Similarly, our measures of instrument control (Figs. 3, 4) displayed changes across trials, but showed no group differences.

One potential reason why 3D observation failed to benefit learning above 2D observation may be due to prior exposure to the task during the baseline test. Firstly, Rohbanfard and Proteau [28] suggest that when physical practice is interspersed with observation, the larger effect of physical practice can wash out the effect of different observation conditions. Here, while there was a general benefit of observation, the effect of physical practice may have overwhelmed any subtle differences between our groups. Secondly, baseline practice may have provided participants with the essential 3D information about the task, such that when observing the 2D video, the combination of monocular cues and previous experience was sufficient to infer any relevant depth information [12].

A more fundamental reason for the similarity across 2D and 3D groups may lie in the underlying mechanism of observational learning. Research into the putative human mirror neuron system [16], a group of motor neurons which fire similarly during produced and observed action, has suggested that observational learning is achieved by the direct simulation of an action in cortical areas of the observer. In effect, the observer is able to practice the movement without carrying it out. This explanation stresses that the key information obtained from observational learning is a direct mapping of how to execute the action [29]. For instance, Meltzoff [30] showed infants choose to turn a light off with their head after seeing the same behaviour in a model. From this perspective, depth perception might be crucial to obtain information about relative bodily (or surgical instrument) movements in 3D space, but in the current study this benefit of 3D information was not observed.

A contrasting explanation of observational learning emphasises that the action patterns acquired by learners through observation may be more dependent on the nature of the task and its surrounding constraints than a direct replication of limb positions [1]. Hodges et al. point to the importance of the end effector (here the surgical instrument) in observational learning, suggesting that the understanding of the goal drives learning, particularly in early skill acquisition. Consequently, the 3D video would provide no additional information regarding the goal and constraints of the task, and therefore would be unlikely to provide any benefit. Research from point-light displays, where human motion is represented by a series lights against a dark background, illustrates that much learning can occur from relatively simple models [31, 32]. Similarly, no performance benefit has been found for watching live versus video models [28] despite enhanced motor cortex activation in live observation [33, 34].

The current findings have important implications for the efficacy of operating room training scenarios based on observing the expert surgeon [25]. During much in vivo training, the operator utilises the 3D view from the robotic system, while the trainee observes the procedure on a 2D slave monitor. As discussed, the surgeon may benefit from additional depth information [14, 27], but based on the current findings, it is unlikely that the trainee is at a disadvantage in being excluded from the 3D view. As identified by Hodges et al. [1] the trainee may acquire key information regarding the end-goal of movements, procedural steps and task constraints entirely successfully from the 2D interface. Therefore, these findings suggest that 3D technology for observing the master surgeon may provide little benefit for the trainee.

Moving forward, further investigation of 3D action observation is required as depth information may benefit task learning when it is informative with regard to the constraints of the task, or the intentions of the model [1]. Additionally, future studies may wish to use an observational learning intervention before any practice has occurred, to prevent learners obtaining prior depth information. Tasks that involve faster or more complex movements, requiring more pre-programming and less online control might also show a stronger 3D effect, as depth cues cannot so easily moderate action during performance. One limitation to consider when interpreting the current results is the use of medical students, who may not represent typical surgical trainees. In order to generalise more effectively, future work may wish to extend these findings to surgical trainees, and compare robotic with laparoscopic tasks.

## Conclusions

The current findings suggest that 3D observation provides no additional benefit to skill learning above 2D observation for early stage robotic skills. However, given the benefits of 3D minimally invasive systems [12, 27] and the preference of teachers and learners for 3D simulators [35], methods for utilising depth information in action observation warrant continued investigation.
